# Personalized ICU mortality assessment by interpretable machine learning algorithms in patients with sepsis combined lung cancer: a population-based study and an external validation cohort

**DOI:** 10.3389/fonc.2025.1661212

**Published:** 2025-10-01

**Authors:** Hongjie Tang, Hairong Hao, Yue Han

**Affiliations:** ^1^ Department of Critical Care Medicine, Xuzhou Central Hospital, Xuzhou, Jiangsu, China; ^2^ Department of Endocrinology and Metabolism, Huai’an Hospital Affiliated to Xuzhou Medical University and Huai’an Second People’s Hospital, Huai’an, Jiangsu, China

**Keywords:** machine learning, sepsis, lung cancer, ICU mortality, MIMIV-IV

## Abstract

**Purpose:**

Sepsis is a leading cause of mortality, especially among immunocompromised patients with lung cancer. We aimed to establish machine learning (ML) based model to accurately forecast ICU mortality in patients with sepsis combined lung cancer.

**Methods:**

We incorporated patients with sepsis combined lung cancer from Medical Information Mart for Intensive Care IV (MIMIC IV) database. Univariate and multivariate logistic analysis were employed to select variables. Recursive Feature Elimination (RFE) method based on 6 ML algorithms was used for feature selection. We harnessed 13 ML algorithms to construct prediction model, which were assessed by area under the curve (AUC), accuracy, sensitivity, specificity, precision, cross-entropy and Brier scores. The best ML model was constructed to predict ICU mortality, and the predictive results were interpretated by SHapley Additive exPlanations (SHAP) framework.

**Results:**

A sum of 1096 lung cancer patients combined sepsis from MIMIC IV database and 251 patients from the external validation set were included. We utilized 13 clinical variables to establish prediction model for ICU mortality. CatBoost model was identified as the prime prediction model with the highest AUC in the training (0.931 [0.921, 0.945]), internal validation (0.698 [0.673, 0.724]) and external validation (0.794 [0.725, 0.879]) cohorts. Oxford Acute Severity of Illness Score (OASIS) had the greatest influence on ICU mortality according to SHAP interpretation.

**Conclusions:**

Our ML models demonstrate excellent accuracy and reliability, facilitating more rigorous personalized prognostic forecast to lung cancer patients combined sepsis.

## Introduction

Sepsis is triggered by an acute infection that induces an excessive and dysregulated immune response in the body, causing multiple organ dysfunctions (1). Each year, approximately 49 million people worldwide are affected by sepsis (2), and about 30% of intensive care unit (ICU) patients are diagnosed with it (3). The mortality rate for sepsis can be as high as 40% (4). Treatment options and outcomes for sepsis patients vary widely due to the differences in infectious agents, individual characteristics, and medical history. Consequently, it is impractical to assess and manage sepsis cases using a single scoring system, and greater attention should be given to the heterogeneity among sepsis patients (5).

Lung cancer remains one of the most prevalent and deadly tumors in the world, with complex challenges in diagnosis, staging, therapy, and future outlook. Accurate diagnosis and staging are crucial for guiding treatment decisions and assessing prognosis. Diagnostic methods involve imaging techniques such as computed tomography (CT) and positron emission tomography (PET), along with histopathological examination and the use of molecular markers to identify specific mutations (e.g., EGFR, ALK) (6). Molecular typing of lung carcinoma, particularly the distinction between small cell lung cancer (SCLC) and non-small cell lung cancer (NSCLC), has further refined treatment approaches, enabling targeted therapies that improve patient outcomes (7). Treatment strategies for lung cancer have evolved significantly, transitioning from conventional chemotherapy and radiation to more individualized approaches. Targeted therapies and immune checkpoint inhibitors, which harness the body’s immune response against cancer cells, have demonstrated efficacy in NSCLC patients with specific genetic alterations (8). For instance, EGFR inhibitors and ALK inhibitors have shown improved survival in patients with these mutations (9). Meanwhile, immunotherapies such as PD-1 and PD-L1 inhibitors have revolutionized treatment for advanced or metastatic cases by extending survival times, although their efficacy varies widely among patients (10). In clinical practice, diagnosing and treating patients with lung cancer complicated by sepsis presents significant challenges. Patients with lung cancer often have compromised immune function, making them more susceptible to infections that can rapidly progress to sepsis, leading to multi-organ dysfunction and increased mortality (11). The complexity of managing lung cancer with concurrent sepsis stems from factors such as tumor burden, immunosuppression, and the adverse effects of anticancer treatments, which complicate early diagnosis and treatment strategies (12).

Nowadays, nomograms have gained widespread application in predicting tumor mortality (13). However, the sensitivity, specificity and generalizability of the previous models, as well as the existing assessment tools, could be inadequate, highlighting the pressing need for more accurate and specific prognostic prediction methods (14). Machine learning (ML), a branch of artificial intelligence, has garnered increasing popularity owing to its proficiency in managing complex, non-linear relationships, especially when dealing with large datasets and loosely structured data (15). The emergence of big data analytics and ML algorithms has rendered new approaches for identifying risk factors influencing prediction feasible. A number of predictive models utilizing these technologies have demonstrated exceptional performance and are progressively being incorporated into clinical practice (16). Nevertheless, to date, no elaborate model exists for predicting ICU mortality in patients with sepsis complicated by lung cancer, underscoring the necessity for the construction and verification of a new ML model for risk stratification. To the best of our knowledge, our research represents the first endeavor to construct and verify a predictive model employing multiple ML algorithms for ICU mortality prediction in patients with sepsis and lung cancer. Our model harnesses extensive population information and the competence of ML, thereby providing an individual predictive model that can help clinicians in meticulously appraising the ICU mortality risk of septic patients with lung cancer.

## Materials and methods

### Data collection and study population

**Figure 1:**
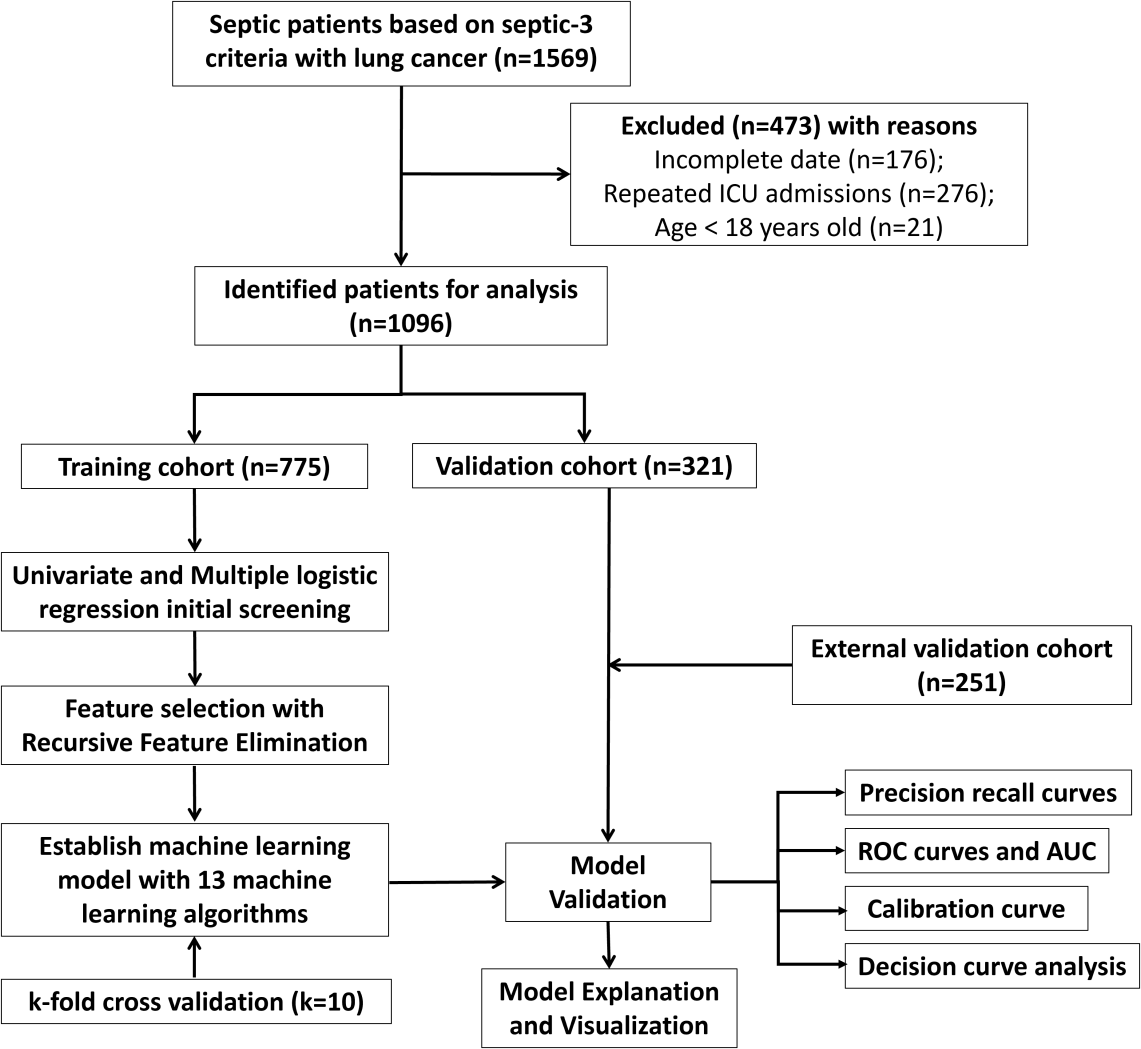
The workflow diagram for study design and patient screening.

The MIMIC-IV database is a publicly available resource containing records of over 76,000 ICU admissions at Beth Israel Deaconess Medical Center in Boston, Massachusetts, USA, from 2008 to 2019. It provides detailed data for every admission, involving laboratory results, vital signs, medications, and discharge status (17). Patients from Xuzhou Central Hospital and Huai’an Hospital Affiliated to Xuzhou Medical University were included to form an external validation set. The research was conducted based on the guidelines of the Declaration of Helsinki and was approved by the Ethics Committee of Xuzhou Central Hospital and Huai’an Hospital Affiliated to Xuzhou Medical University. Informed consent was acquired from patients involved in our research. The flowchart of the patient selection procedure is displayed in **Figure 1**. Inclusion criteria comprised individuals diagnosed as lung cancer and sepsis based on International Classification of Diseases (Ninth Revision code), as well as aged over eighteen years at the time admitted by ICU. Sepsis diagnosis was conducted according to sepsis definition 3.0 (18). Exclusion criteria comprised patients with repeated ICU admissions except for the first time or clinical variables with more than 50% missing data. Clinical information of septic lung cancer patients in MIMIC IV database included the following (listed in **Table 1**): (1) demographics (age and sex); (2) tumor stages, with distant metastasis defined by the American Joint Committee on Cancer 8th edition; (3) chronic conditions such as hypertension or diabetes; (4) organ functions assessed by the Sequential Organ Failure Assessment (SOFA) score; (5) laboratory tests. Vital signs and laboratory results from the first 24 hours of ICU admission were included. Missing information was tackled with multiple imputation by chained equations (MICE). The study’s endpoints were ICU death or safe discharge. Raw data extraction via Navicat for SQL Server was processed using R software. We determined the minimum sample size needed for an external validation cohort by formula of Riley et al. (19).

**Table 1:** Clinicopathological characteristics of septic patients with lung cancer from MIMIC IV database in the ICU Non-Mortality group and ICU Mortality group.

Variable	ICU Non-Mortality N = 851	ICU Mortality N = 245	p.overall
Age	68.4 (12.3)	68.4 (12.8)	0.968
Gender:			0.095
Female	384 (45.1%)	126 (51.4%)	
Male	467 (54.9%)	119 (48.6%)	
Race:			0.066
Asian	50 (5.88%)	17 (6.94%)	
Black	90 (10.6%)	30 (12.2%)	
Other	108 (12.7%)	45 (18.4%)	
White	603 (70.9%)	153 (62.4%)	
Marital status:			0.418
Divorced	65 (7.64%)	14 (5.71%)	
Married	440 (51.7%)	119 (48.6%)	
Null	36 (4.23%)	15 (6.12%)	
Single	198 (23.3%)	66 (26.9%)	
Widowed	112 (13.2%)	31 (12.7%)	
Hypertension:			0.183
No	510 (59.9%)	159 (64.9%)	
Yes	341 (40.1%)	86 (35.1%)	
Diabetes:			0.161
No	693 (81.4%)	189 (77.1%)	
Yes	158 (18.6%)	56 (22.9%)	
Cardiac arrhythmia:			0.599
No	807 (94.8%)	235 (95.9%)	
Yes	44 (5.17%)	10 (4.08%)	
Metastatic Cancer:			0.047
No	480 (56.4%)	120 (49.0%)	
Yes	371 (43.6%)	125 (51.0%)	
Weight	74.9 (21.6)	73.1 (23.4)	0.269
SOFA	4.65 (2.93)	6.89 (3.66)	<0.001
Acute Physiology Score III	47.0 (18.2)	63.6 (23.0)	<0.001
SIRS	2.71 (0.86)	2.97 (0.80)	<0.001
SAPS II	43.2 (12.8)	53.2 (15.7)	<0.001
OASIS	32.8 (7.81)	39.6 (8.58)	<0.001
Glasgow Coma Scale	13.6 (2.54)	13.1 (3.33)	0.06
WBC	11.9 (7.88)	14.8 (11.2)	<0.001
RBC	3.44 (0.67)	3.38 (0.63)	0.155
Platelet	235 (133)	239 (148)	0.686
Hemoglobin	10.1 (1.92)	9.76 (1.77)	0.013
RDW	16.0 (2.54)	16.7 (2.48)	<0.001
Hematocrit	31.0 (5.65)	30.5 (5.25)	0.155
Sodium	137 (4.65)	137 (5.83)	0.028
Potassium	4.24 (0.59)	4.47 (0.70)	<0.001
Calcium	8.32 (0.84)	8.32 (1.06)	0.964
Chloride	103 (5.99)	101 (6.76)	0.015
Glucose	137 (54.7)	140 (51.7)	0.553
Anion gap	14.7 (3.51)	15.6 (4.04)	0.001
PH	7.37 (0.07)	7.34 (0.09)	<0.001
pCO2	43.5 (12.0)	46.9 (13.6)	0.001
pO2	108 (69.2)	103 (51.8)	0.287
Lactate	1.98 (1.48)	2.79 (2.24)	<0.001
Total CO2	26.0 (6.07)	25.9 (6.82)	0.72
PT	16.2 (9.10)	17.3 (8.03)	0.073
PTT	36.2 (18.8)	39.7 (21.0)	0.029
INR	1.49 (1.03)	1.60 (0.81)	0.1
Urea nitrogen	26.7 (20.9)	32.2 (21.4)	0.001
Creatinine	1.29 (1.17)	1.34 (1.09)	0.491
Heart rate	90.6 (16.2)	96.5 (17.3)	<0.001
Non invasive systolic blood pressure	114 (20.6)	126 (263)	0.502
Non invasive diastolic blood pressure	63.2 (10.6)	61.9 (11.1)	0.099
Non invasive mean blood pressure	75.8 (12.1)	72.9 (10.7)	<0.001
Oxygen saturation	96.3 (2.12)	95.6 (3.08)	<0.001
Temperature	36.8 (1.43)	36.8 (0.51)	0.428
Hospital day	12.4 (11.1)	6.70 (6.06)	<0.001
ICU day	4.28 (5.32)	5.58 (5.41)	0.001
Hospital survival day	149 (295)	6.23 (6.06)	<0.001
ICU survival day	148 (295)	4.92 (5.42)	<0.001

### Feature selection

In our initial analysis, clinic variables with a significance level of P < 0.05 in both univariate and multivariate logistic analyses within the training dataset were selected for feature selection. We then applied Recursive Feature Elimination (RFE) according to six ML approaches, namely categorical boosting (CatBoost), random forest (RF), support vector machine (SVM), extreme gradient boosting (XGB), decision tree (DT), and gradient boosting machine (GBM), coupled with 10-fold cross-validation to select the clinic variables. The RFE process involves iteratively building models and ranking features by their importance, systematically removing the least significant ones to generate a comprehensive feature ranking (20). A random seed of “123” was determined for our analysis. Subsequently, the Robust Rank Aggregation (RRA) algorithm was employed to consolidate the feature importance rankings from the six ML algorithms utilized in RFE, yielding a comprehensive ranking of all factors (21). Following the selection of key features, we proceed to the model development stage.

### Development and verification of the predictive model for ICU mortality

To develop the ML model, we utilized thirteen ML algorithms, involving CatBoost, RF, SVM, XGB, DT, GBM, k-nearest neighbor (KNN), logistic regression (LR), naive bayes classifier (NBC), linear discriminant analysis (LDA), quadratic discriminant analysis (QDA), neural network (NNET) and generalized linear model (GLM) to forecast ICU mortality with “mlr3” R package (22). This method facilitated the comparison of model performances and the selection of the optimal predictive model. To address class imbalance, which can significantly distort performance metrics, we applied the Synthetic Minority Over-sampling Technique (SMOTE) during model training (23). We then enhanced our methodology by conducting nested resampling, involving a two-tiered k-fold cross-validation process: one for hyperparameter tuning and another for model selection. Additionally, we conducted a 1000-evaluation random searching within a 10-fold cross-validation framework, repeating five times in every model. The best model was selected based on the highest Area Under the Curve (AUC) and the lowest Brier score, while ensuring a well-calibrated curve. Internal and external validation was performed using 10-fold cross-validation. The Precision-Recall Curve (PRC) was used to assess the performances of classification models on imbalanced data. The calibration curve evaluated the model’s discriminative ability, and Decision Curve Analysis (DCA) was conducted to validate the clinical benefits of the ML model using the “runway” R package (https://github.com/ML4LHS/runway). The importance of every factor was quantified by calculating its mean contribution to the AUC as a percentage relative to the full model using the “DALEX” R package (24). SHapley Additive exPlanations (SHAP) values were employed to demonstrate the predictions of the optimal model and to clarify the black-box ML framework using the “shapviz” R package (https://github.com/ModelOriented/shapviz) (25).

## Results

### Demographic composition and baseline data

A sum of 1096 lung cancer patients combined with sepsis from MIMIC IV database and 251 patients from Xuzhou Central Hospital and Huai’an Hospital Affiliated to Xuzhou Medical University were involved. We separated patients in MIMIC IV cohort randomly into training and internal testing cohorts with a 7:3 ratio, respectively. Meanwhile, patients in Xuzhou Central Hospital and Huai’an Hospital Affiliated to Xuzhou Medical University were involved as the external testing cohort. For patients in MIMIC IV cohort, 854 cases (77.65%) were alive, while 245 cases (22.35%) suffered ICU mortality (**Table 1**). More clinic data of the training and two testing cohorts can be found in **Table 2**. In the training, internal validation and external validation sets, the ICU mortality was 161 (20.8%), 84 (26.2%) and 40 (15.9%) (**Table 2**). The detailed selection process of patients in MIMIC IV cohort is displayed in **Figure 1**.

**Table 2:** Clinicopathological characteristics of septic patients with lung cancer in the training, internal validation and external validation cohorts.

Variable	Training Cohort N = 775	Validation Cohort N = 321	External Validation Cohort N = 251	p.overall
Age	68.2 (12.3)	68.9 (12.6)	68.2 (12.4)	0.719
Gender:				0.932
Female	360 (46.5%)	150 (46.7%)	120 (47.8%)	
Male	415 (53.5%)	171 (53.3%)	131 (52.2%)	
Race:				<0.001
Asian	49 (6.32%)	18 (5.61%)	251 (100%)	
Black	88 (11.4%)	32 (9.97%)	0 (0.00%)	
Other	104 (13.4%)	49 (15.3%)	0 (0.00%)	
White	534 (68.9%)	222 (69.2%)	0 (0.00%)	
Marital status:				0.455
Divorced	61 (7.87%)	18 (5.61%)	18 (7.17%)	
Married	385 (49.7%)	174 (54.2%)	125 (49.8%)	
Null	35 (4.52%)	16 (4.98%)	13 (5.18%)	
Single	200 (25.8%)	64 (19.9%)	62 (24.7%)	
Widowed	94 (12.1%)	49 (15.3%)	33 (13.1%)	
Hypertension:				0.988
No	474 (61.2%)	195 (60.7%)	154 (61.4%)	
Yes	301 (38.8%)	126 (39.3%)	97 (38.6%)	
Diabetes:				0.844
No	624 (80.5%)	258 (80.4%)	206 (82.1%)	
Yes	151 (19.5%)	63 (19.6%)	45 (17.9%)	
Cardiac arrhythmia:				0.221
No	742 (95.7%)	300 (93.5%)	241 (96.0%)	
Yes	33 (4.26%)	21 (6.54%)	10 (3.98%)	
Metastatic Cancer:				0.42
No	433 (55.9%)	167 (52.0%)	132 (52.6%)	
Yes	342 (44.1%)	154 (48.0%)	119 (47.4%)	
Weight	74.1 (22.0)	75.4 (22.0)	73.7 (24.2)	0.598
SOFA	5.07 (3.18)	5.35 (3.38)	5.18 (3.31)	0.417
Acute Physiology Score III	50.2 (19.9)	52.0 (22.0)	51.4 (20.4)	0.391
SIRS	2.75 (0.84)	2.81 (0.88)	2.71 (0.84)	0.359
SAPS II	45.1 (14.2)	46.3 (13.9)	46.3 (15.8)	0.288
OASIS	34.1 (8.52)	34.7 (8.34)	34.5 (8.93)	0.564
Glasgow Coma Scale	13.5 (2.75)	13.5 (2.72)	13.2 (3.07)	0.484
WBC	12.5 (8.71)	12.7 (9.09)	12.1 (8.80)	0.738
RBC	3.42 (0.65)	3.45 (0.69)	3.44 (0.68)	0.718
Platelet	237 (135)	232 (139)	230 (125)	0.698
Hemoglobin	9.97 (1.88)	10.1 (1.92)	9.95 (1.87)	0.483
RDW	16.2 (2.54)	16.1 (2.54)	16.3 (2.47)	0.547
Hematocrit	30.8 (5.54)	31.3 (5.62)	30.6 (5.59)	0.274
Sodium	137 (5.03)	138 (4.74)	137 (4.82)	0.21
Potassium	4.29 (0.64)	4.28 (0.57)	4.33 (0.66)	0.594
Calcium	8.34 (0.89)	8.28 (0.88)	8.35 (0.99)	0.605
Chloride	102 (6.34)	103 (5.82)	102 (5.88)	0.674
Glucose	137 (55.0)	139 (51.7)	137 (49.3)	0.878
Anion gap	14.8 (3.62)	15.0 (3.74)	14.7 (3.64)	0.552
PH	7.36 (0.08)	7.36 (0.08)	7.37 (0.08)	0.631
pCO2	44.5 (12.1)	44.3 (13.5)	44.5 (12.4)	0.971
pO2	106 (65.2)	107 (64.5)	111 (70.6)	0.642
Lactate	2.16 (1.74)	2.27 (1.79)	2.14 (1.76)	0.648
Total CO2	26.1 (6.05)	25.6 (6.77)	26.3 (6.12)	0.52
PT	16.7 (9.51)	16.0 (7.13)	17.5 (12.8)	0.224
PTT	36.7 (18.3)	37.9 (21.6)	37.6 (20.7)	0.634
INR	1.54 (1.08)	1.46 (0.68)	1.65 (1.58)	0.199
Urea nitrogen	28.1 (21.6)	27.6 (20.0)	27.6 (21.0)	0.89
Creatinine	1.29 (1.15)	1.32 (1.15)	1.34 (1.38)	0.816
Heart rate	91.4 (16.2)	93.2 (17.6)	89.7 (16.4)	0.041
Non invasive systolic blood pressure	114 (20.6)	125 (232)	112 (17.2)	0.262
Non invasive diastolic blood pressure	63.0 (10.6)	62.8 (10.9)	62.4 (10.2)	0.797
Non invasive mean blood pressure	75.1 (10.8)	75.2 (14.1)	74.5 (10.5)	0.736
Oxygen saturation	96.2 (2.33)	96.1 (2.51)	96.3 (2.15)	0.553
Temperature	36.8 (1.50)	36.8 (0.45)	36.7 (1.27)	0.417
Hospital day	11.2 (10.9)	11.0 (9.38)	11.3 (14.0)	0.967
ICU day	4.48 (5.53)	4.79 (4.92)	4.60 (6.23)	0.7
Hospital survival day	113 (269)	103 (235)	112 (214)	0.859
ICU survival day	112 (269)	102 (235)	110 (214)	0.858
ICU mortality:				0.011
No	614 (79.2%)	237 (73.8%)	211 (84.1%)	
Yes	161 (20.8%)	84 (26.2%)	40 (15.9%)	

### Feature selection of the predictive model

**Figure 2:**
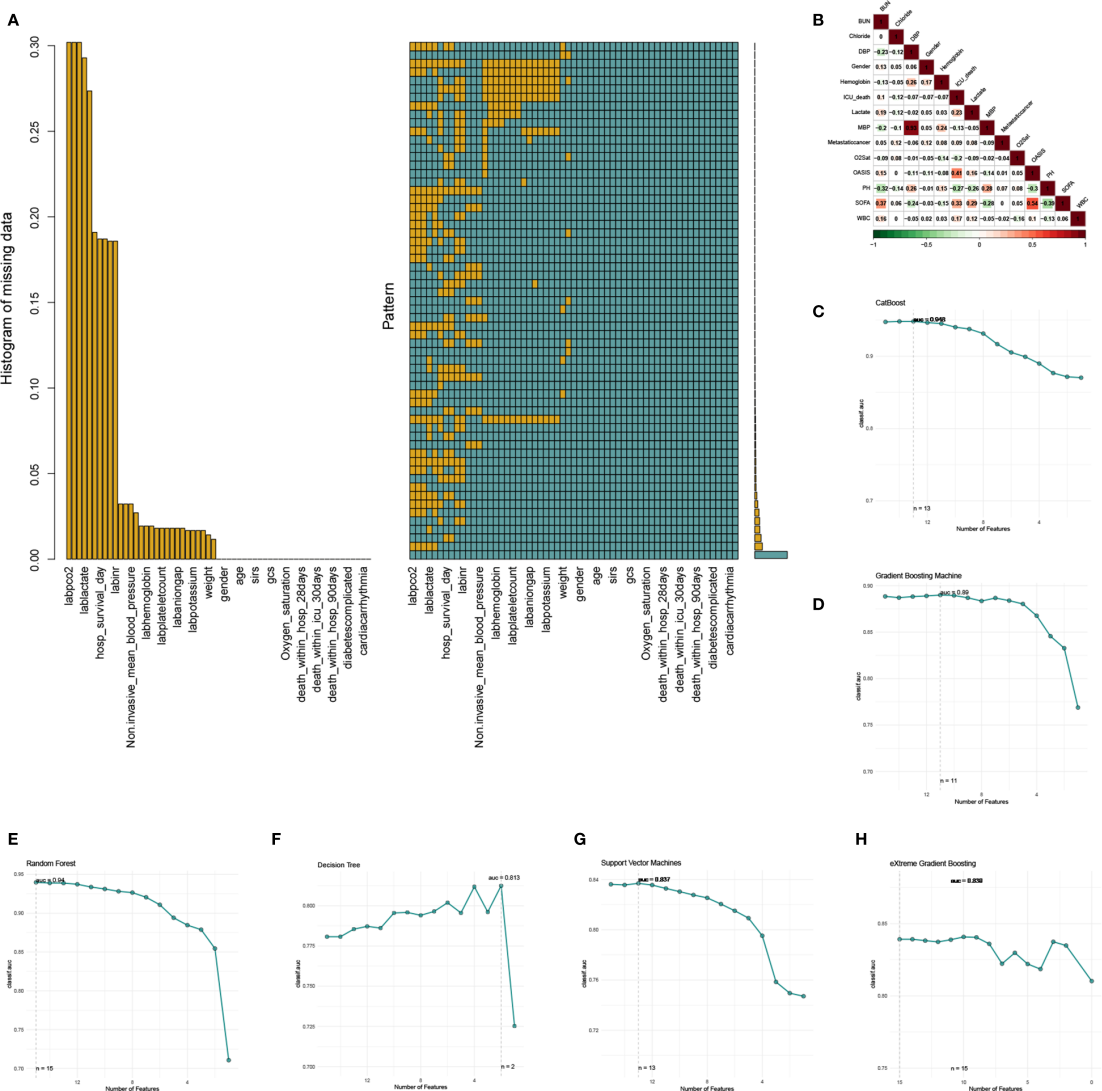
The process of data filtering and feature selection. **(A)** Visualization of missing data patterns. **(B)** The heatmap of Spearman’s correlation analysis of the clinic variables with ICU mortality. The correlation index ranges from -1.0 to 1.0, with a brighter color indicating a stronger correlation. **(C-H)** Feature selection process with Recursive Feature Elimination (RFE) method based on six ML algorithms (CatBoost, GBM, RF, DT, SVM, and XGB).

We used the multiple imputation by chained equations (MICE) method to address the missing information in our patient data from MIMIC IV database (**Figure 2A**). Ultimately, five imputed datasets were created, and Rubin’s rules were utilized to amalgamate the final analytical outcomes (**Supplementary Figure 1**). Drawing from our clinical expertise, these clinic variables were chosen for subsequent logistic regression analysis (**Table 3**), with variables with a correlation coefficient exceeding 0.6 excluded (**Figure 2B**). Thereafter, univariate and multivariate logistic regression analyses were conducted within the training cohort to identify the salient variables predictive of ICU mortality. We then discovered that Urea nitrogen (BUN, OR 1.19 (1.08-1.25), p = 0.003), Chloride (OR 0.94 (0.91-0.96), p < 0.001), Diastolic blood pressure (DBP, OR 0.99 (0.98-1), p = 0.035), Gender (OR 0.69 (0.5-0.97), p = 0.031), Hemoglobin (OR 0.84 (0.77-0.93), p < 0.001), Lactate (OR 1.2 (1.08-1.35), p = 0.001), Mean blood pressure (MBP, OR 1.01 (1-1.04), p = 0.031), Metastatic cancer (OR 2.14 (1.52-3.02), p < 0.001), Oxygen saturation (OR 0.72 (0.66-0.78), p < 0.001), OASIS (OR 1.1 (1.07-1.13), p < 0.001), PH (OR 0.01 (0-0.09), p < 0.001), SOFA (OR 1.13 (1.06-1.2), p < 0.001), WBC (OR 1.04 (1.02-1.06), p < 0.001), Age (OR 1.02 (1-1.03), p = 0.031) and Acute Physiology Score III (OR 1.03 (1.01-1.05), p = 0.009) were exactly important to forecast ICU mortality, with significance (p < 0.05, **Table 3**). Correlation analysis revealed that OASIS is the most influential variable associated with ICU mortality (**Figure 2B**). Subsequently, we employed RFE leveraging six ML algorithms (GBM, SVM, RF, DT, XGB and CatBoost), coupled with 1–fold cross-validation to refine the clinical variables (**Figures 2C-H**). The RFE process identified the optimal feature set using the CatBoost algorithm, which retained thirteen variables and achieved the highest AUC of 0.948 (**Figure 2C**). The RRA algorithm was then applied to generate a comprehensive ranking of the clinical variables across the six ML algorithms, with OASIS emerging as the most vital (**Supplementary Table 1**). These thirteen variables, selected by RFE, were subsequently incorporated into the subsequent model establishment procedures. (**Supplementary Table 1**).

**Table 3:** Univariate and multivariate logistics analysis of septic patients with lung cancer for predicting ICU mortality in the training cohort.

Variable	Univariable logistic analysis		Multivariate logistic analysis	
term	OR (95%CI)	p.value	OR (95%CI)	p.value
Age	1.01 (1-1.02)	0.044	1.02 (1-1.03)	0.031
Gender: Female	Reference			
Male	0.77 (0.59-1)	0.050	0.69 (0.5-0.97)	0.031
Race: Asian	Reference			
Black	1.53 (0.92-2.51)	0.099	0.63 (0.21-1.95)	0.418
Other	1.91 (1.21-2.99)	0.005	1.38 (0.49-3.99)	0.546
White	1.16 (0.83-1.64)	0.383	0.68 (0.27-1.8)	0.432
Marital status: Divorced	Reference			
Married	1.05 (0.62-1.86)	0.862		
Null	1.72 (0.81-3.65)	0.156		
Single	1.38 (0.79-2.5)	0.269		
Widowed	1.25 (0.68-2.37)	0.484		
Hypertension: No	Reference			
Yes	0.81 (0.62-1.07)	0.137		
Diabetes: No	Reference			
Yes	1.29 (0.93-1.76)	0.120		
Cardiac arrhythmia: No	Reference			
Yes	0.85 (0.43-1.57)	0.629		
Metastatic Cancer: No	Reference			
Yes	1.28 (0.99-1.67)	0.063	2.14 (1.52-3.02)	<0.001
Weight	0.99 (0.99-1)	0.048		
SOFA	1.2 (1.15-1.25)	<0.001	1.13 (1.06-1.2)	<0.001
Acute Physiology Score III	1.04 (1.03-1.04)	<0.001	1.03 (1.01-1.05)	0.009
SIRS	1.48 (1.26-1.75)	<0.001	1 (0.72-1.39)	1
SAPS II	1.05 (1.04-1.05)	<0.001	1.01 (0.98-1.04)	0.515
OASIS	1.1 (1.08-1.12)	<0.001	1.1 (1.07-1.13)	<0.001
Glasgow Coma Scale	0.95 (0.92-1)	0.037	0.97 (0.93-1.06)	0.061
WBC	1.03 (1.02-1.05)	<0.001	1.04 (1.02-1.06)	<0.001
RBC	0.89 (0.72-1.08)	0.249		
Platelet	1 (1-1)	0.252		
Hemoglobin	0.92 (0.85-0.98)	0.018	0.84 (0.77-0.93)	<0.001
RDW	1.1 (1.04-1.15)	<0.001	1.15 (0.94-1.27)	0.067
Hematocrit	0.98 (0.96-1.01)	0.167		
Sodium	0.96 (0.94-0.99)	0.004	0.92 (0.83-1.02)	0.135
Potassium	1.72 (1.4-2.1)	<0.001	1.47 (0.94-2.4)	0.055
Calcium	1.08 (0.94-1.25)	0.285		
Chloride	0.96 (0.94-0.99)	<0.001	0.94 (0.91-0.96)	<0.001
Glucose	1 (1-1)	0.543		
Anion gap	1.06 (1.03-1.1)	<0.001	1.02 (0.92-1.14)	0.683
PH	0 (0-0.02)	<0.001	0.01 (0-0.09)	<0.001
pCO2	1.02 (1.01-1.03)	<0.001	1.08 (0.94-1.23)	0.061
pO2	1 (1-1)	0.250		
Lactate	1.25 (1.15-1.36)	<0.001	1.2 (1.08-1.35)	0.001
Total CO2	1 (0.98-1.02)	0.942		
PT	1.01 (1-1.02)	0.132		
PTT	1.01 (1-1.01)	0.016	1 (0.99-1.01)	0.987
INR	1.07 (0.95-1.2)	0.257		
Urea nitrogen	1.01 (1-1.02)	<0.001	1.19 (1.08-1.25)	0.003
Creatinine	1 (0.89-1.11)	0.964		
Heart rate	1.02 (1.02-1.03)	<0.001	1.01 (0.99-1.02)	0.366
Non invasive systolic blood pressure	1 (1-1)	0.311		
Non invasive diastolic blood pressure	0.98 (0.97-1)	0.009	0.99 (0.98-1)	0.035
Non invasive mean blood pressure	0.98 (0.97-0.99)	0.002	1.01 (1-1.04)	0.031
Oxygen saturation	0.88 (0.83-0.92)	<0.001	0.72 (0.66-0.78)	<0.001
Temperature	1.08 (0.95-1.37)	0.425		

### Construction and verification of ML model for ICU mortality

**Figure 3:**
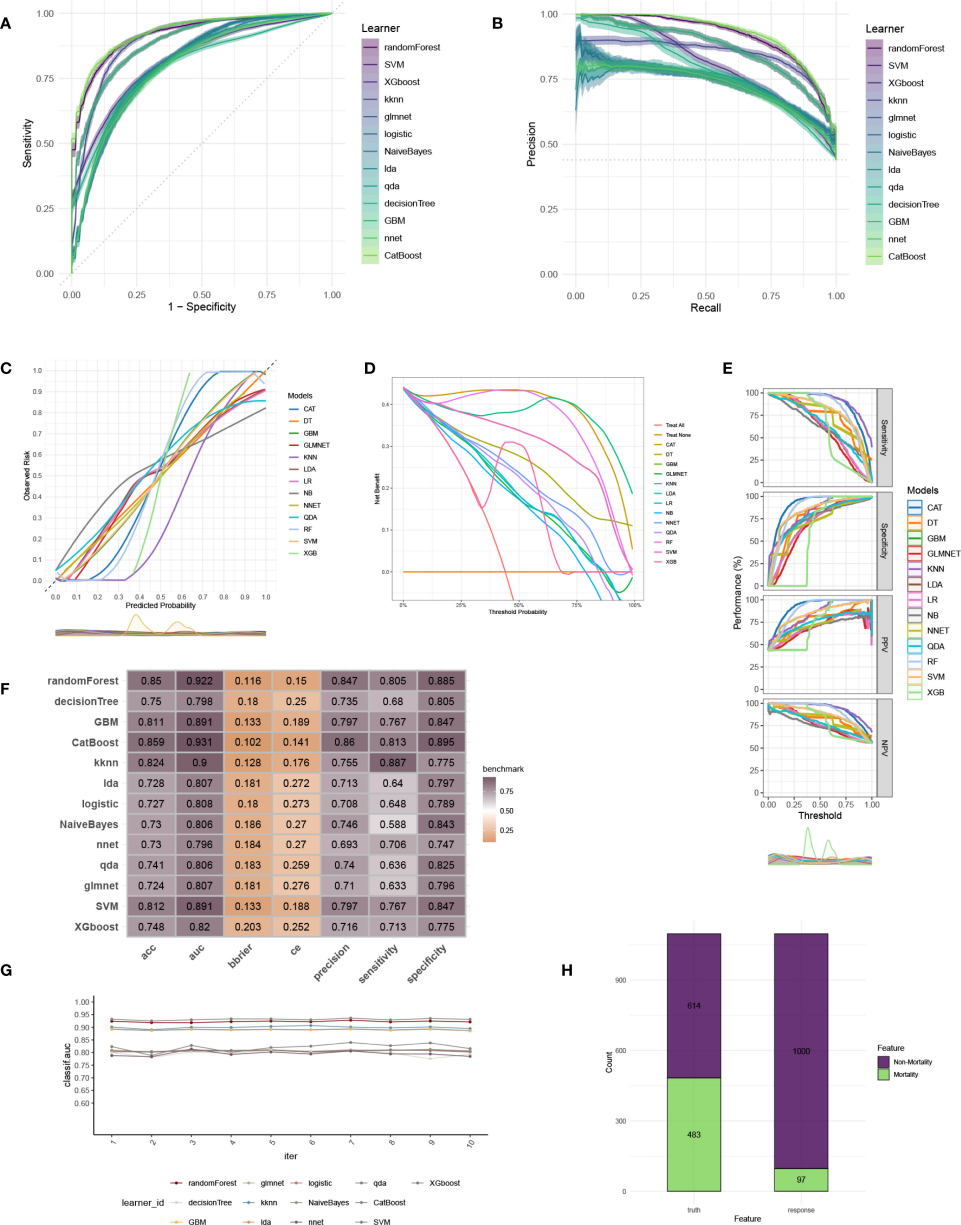
Establishment and evaluation of the ML models in the training set. **(A)** ROC curves of different ML models in the training set. **(B)** PR curves of different ML models in the training set. **(C)** Calibration curves of different ML models in the training set. **(D)** DCA curves of different ML models in the training set. **(E)** The curves of sensitivity, specificity, PPV and NPV of the 13 ML models in the training set. **(F)** The performance of 13 ML models in terms of AUC, accuracy, sensitivity, specificity, precision, cross-entropy and Brier scores in the training set. **(G)** Ten-fold cross-validation results of different ML models in the training set. **(H)** Confusion matrix of the best ML model in the training set. ML, machine learning; CAT, categorical boosting; LR, logistic regression; DT, decision tree; RF, random forest; XGB, extreme gradient boosting; GBM, gradient boosting machine; NB, Naive Bayes; LDA, linear discriminant analysis; QDA, quadratic discriminant analysis; NNET, neural network; GLMNET, generalized linear models with elastic net regularization; SVM, support vector machine; KNN, k-nearest neighbor.

**Figure 4:**
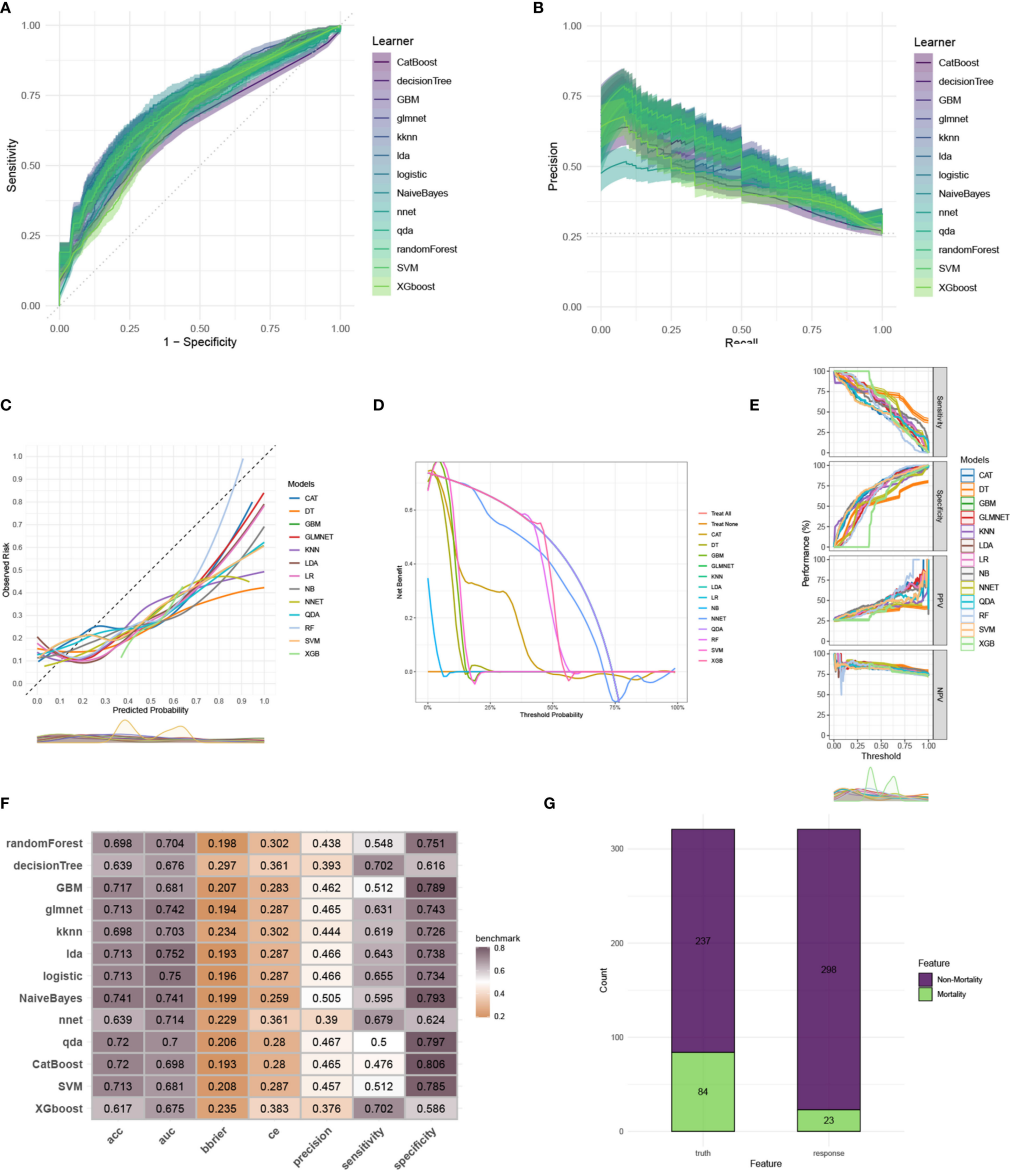
Evaluation of the ML models in the internal validation set. **(A)** ROC curves of different ML models in the internal validation set. **(B)** PR curves of different ML models in the internal validation set. **(C)** Calibration curves of different ML models in the internal validation set. **(D)** DCA curves of different ML models in the internal validation set. **(E)** The curves of sensitivity, specificity, PPV and NPV of the 13 ML models in the training set. **(F)** The performance of 13 ML models in terms of AUC, accuracy, sensitivity, specificity, precision, cross-entropy and Brier scores in the internal validation set. **(G)** Confusion matrix of the best ML model in the internal validation set. ML, machine learning; CAT, categorical boosting; LR, logistic regression; DT, decision tree; RF, random forest; XGB, extreme gradient boosting; GBM, gradient boosting machine; NB, Naive Bayes; LDA, linear discriminant analysis; QDA, quadratic discriminant analysis; NNET, neural network; GLMNET, generalized linear models with elastic net regularization; SVM, support vector machine; KNN, k-nearest neighbor.

**Figure 5:**
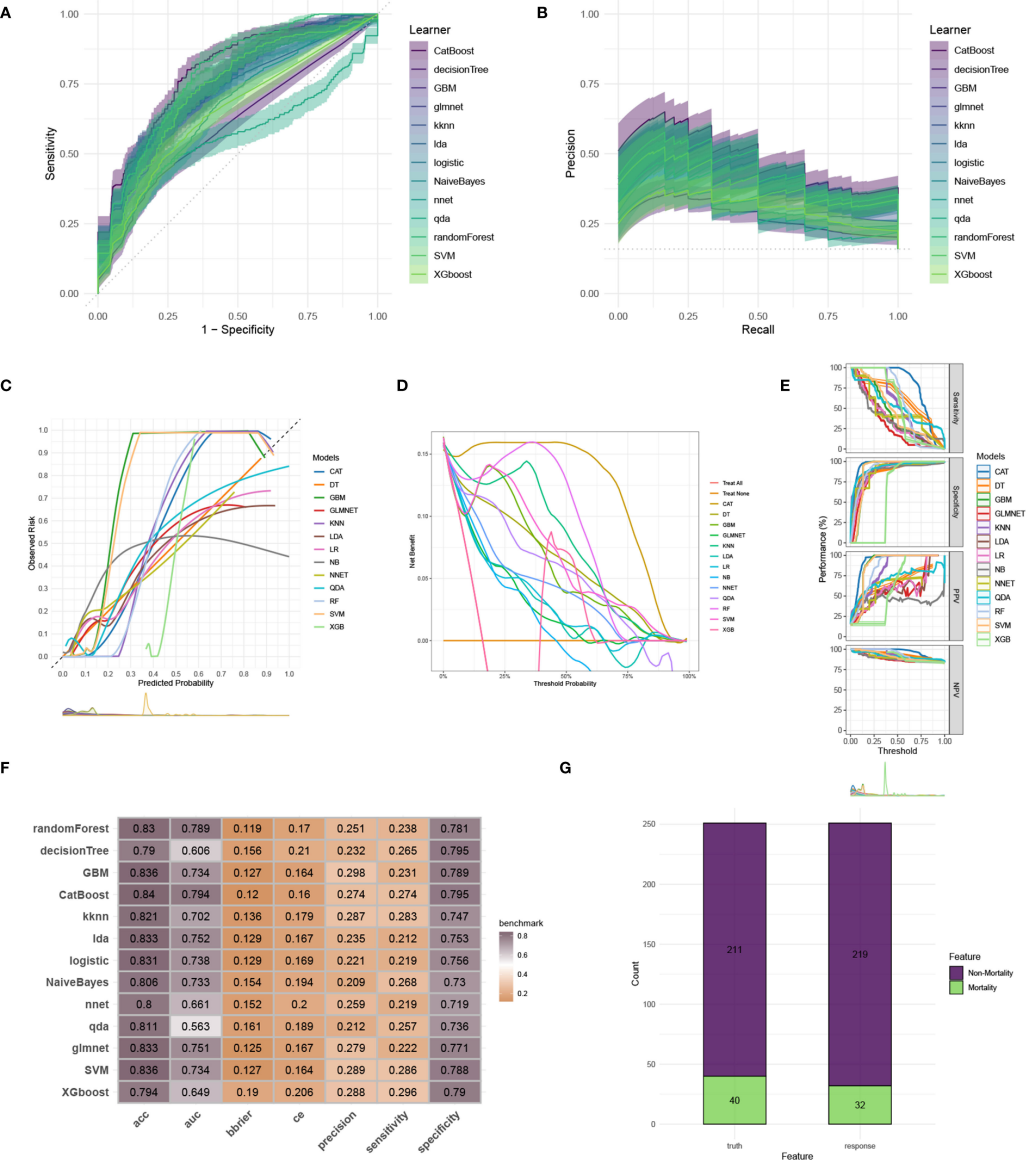
Evaluation of the ML models in the external validation set. **(A)** ROC curves of different ML models in the external validation set. **(B)** PR curves of different ML models in the external validation set. **(C)** Calibration curves of different ML models in the external validation set. **(D)** DCA curves of different ML models in the external validation set. **(E)** The curves of sensitivity, specificity, PPV and NPV of the 13 ML models in the training set. **(F)** The performance of 13 ML models in terms of AUC, accuracy, sensitivity, specificity, precision, cross-entropy and Brier scores in the external validation set. **(G)** Confusion matrix of the best ML model in the external validation set. ML, machine learning; CAT, categorical boosting; LR, logistic regression; DT, decision tree; RF, random forest; XGB, extreme gradient boosting; GBM, gradient boosting machine; NB, Naive Bayes; LDA, linear discriminant analysis; QDA, quadratic discriminant analysis; NNET, neural network; GLMNET, generalized linear models with elastic net regularization; SVM, support vector machine; KNN, k-nearest neighbor.

To construct an accurate model to forecast ICU mortality, we included the thirteen clinic factors (“BUN”, “Chloride”, “DBP”, “Gender”, “Hemoglobin”, “Lactate”, “MBP”, “Metastatic cancer”, “O2Sat”, “OASIS”, “PH”, “SOFA”, “WBC”) selected by RFE based on CatBoost. Totally thirteen ML algorithms, involving CatBoost, RF, SVM, XGB, DT, GBM, KNN, LR, NBC, LDA, QDA, NNET and GLM, were developed using the selected thirteen variables from the training set. Hyperparameter tuning were optimized through 5-fold cross-validation and random searches. The performance of these thirteen models was then assessed in both internal and external validation cohorts. ROC curve analysis indicated that the CatBoost model achieved the highest AUC in the training (0.931 [0.921, 0.945]), internal validation (0.698 [0.673, 0.724]), and external validation (0.794 [0.725, 0.879]) cohorts (**Figures 3A**, **4A**, **5A**). Following hyperparameter tuning via grid search, the optimal hyperparameters for CatBoost were identified as depth, 6; learning_rate, 0.02873998; iterations, 662; 12_leaf_reg, 6.735671. PRC analysis demonstrated the CatBoost model’s effectiveness in managing imbalanced data (**Figures 3B**, **4B**, **5B**). Calibration curves revealed that CatBoost algorithm had the best fitting ability and could accurately predict ICU mortality (**Figures 3C**, **4C**, **5C**). Calibration curves indicated that CatBoost algorithm’s probability predictions are consistent and well-calibrated, and ensured that the risk estimates provided by the model can be trusted to reflect the true likelihood of ICU mortality. DCA curves implied that the CatBoost algorithm had the highest clinical utility and could effectively aid in predicting ICU mortality (**Figures 3D**, **4D**, **5D**). DCA curves indicated that using the CatBoost model to guide clinical decision-making would result in net clinical benefit for patients who are likely to benefit from certain interventions, such as more aggressive treatment or intensive monitoring. The curves of sensitivity, specificity, positive predictive value (PPV), and negative predictive value (NPV) of the thirteen ML algorithms were plotted to extensively identified that the CatBoost algorithm was outperforming in predicting ICU mortality (**Figures 3E**, **4E**, **5E**). Model performance was further evaluated using accuracy, sensitivity, specificity, precision, cross-entropy, and Brier scores, which collectively indicated the robustness of the CatBoost model in predicting ICU mortality (**Figures 3F**, **4F**, **5F**). Tenfold cross-validation in the training cohort also confirmed the superior performance of CatBoost (**Figure 3G**). Confusion matrices highlighted the outstanding predictive capabilities of CatBoost across all three cohorts (**Figures 3H**, **4G**, **5G**). Hence, CatBoost was selected as the optimal model for predicting ICU mortality and model validation was sufficient for proving its capacity.

### Model interpretation

**Figure 6:**
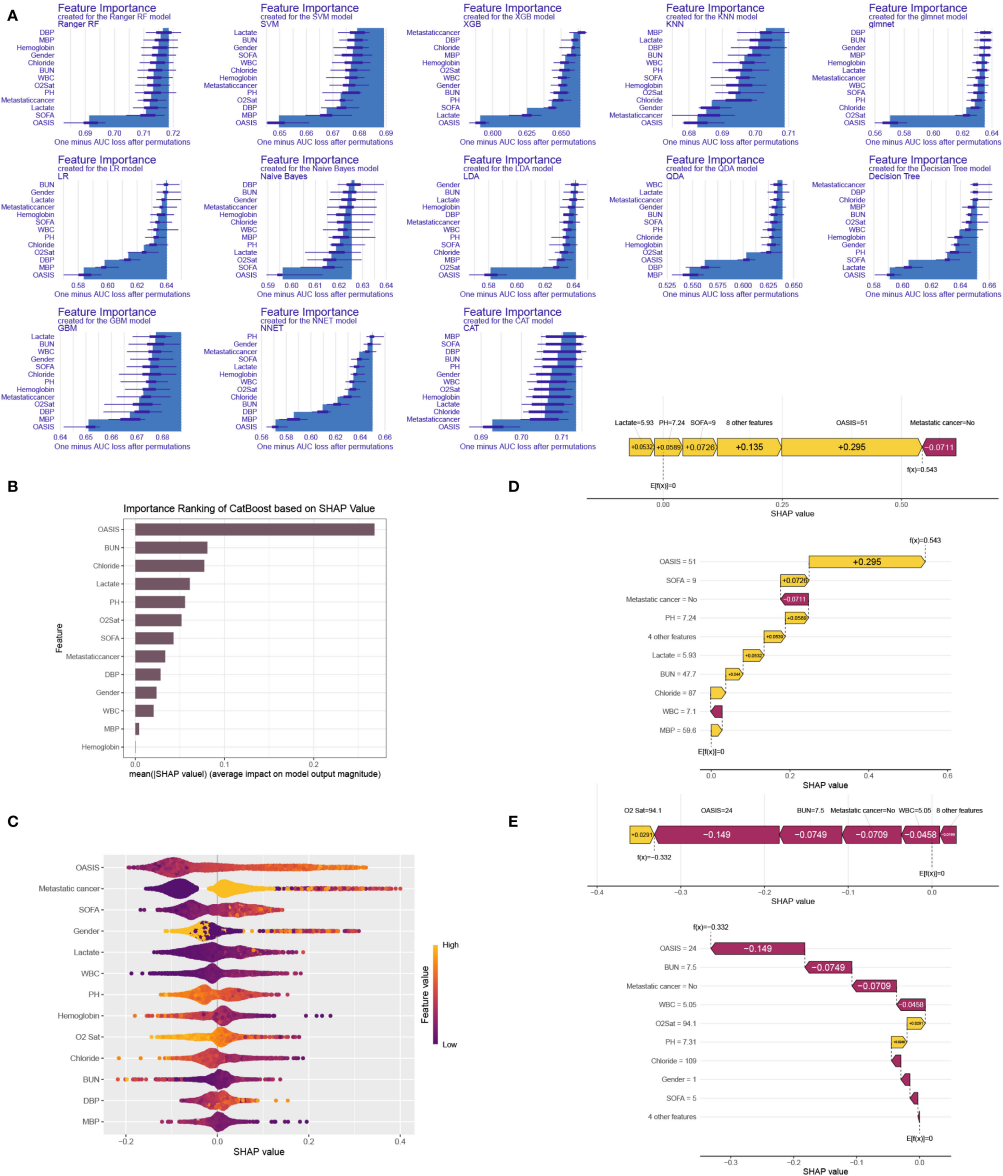
ML model interpretation. **(A)** Importance ranking of features in 13 ML prediction algorithms (CatBoost, RF, NNET, GBM, SVM, KNN, DT and GLM). **(B)** The importance ranking of different variables according to the mean (|SHAP value|) using the optimal CatBoost model. **(C)** The importance ranking of different risk factors with stability and interpretation using the optimal CatBoost model. The higher SHAP value of a feature is given, the higher risk of ICU mortality the patient would have. The yellow part in feature value represents higher value. **(D)** SHAP value explanation in a classical sample with ICU mortality. **(E)** SHAP value explanation in a classical sample without ICU mortality.

We computed and depicted the ranking of every feature importance for every ML model, involving CatBoost, RF, NNET, GBM, SVM, KNN, DT and GLM models (**Figure 6A**). The importance scores were derived from the intrinsic properties of the respective ML algorithms, highlighting that the factor most strongly associated with ICU mortality were predominantly “OASIS”. Afterwards, we employed the SHAP framework to elucidate the CatBoost model. We visualized the variables by their mean absolute SHAP values, which confirmed that “OASIS” was the most influential variable (**Figure 6B**). Additionally, a bee swarm plot illustrated the impact of every clinic variable on ICU mortality (**Figure 6C**). The y-axis represents the magnitude of risk factor, and the x-axis denotes their effect on model output, exactly ICU mortality, as quantified by the SHAP value. The plot revealed that higher OASIS, SOFA, lactate, and WBC levels were related to an elevated risk of ICU mortality, and patients with metastatic cancer were prone to suffer ICU mortality. To demonstrate model interpretability, we examined two representative patients. SHAP values were utilized to assess the influence of each feature on the model’s predictions. In our study, low SHAP values indicated a reduced likelihood of ICU mortality, whereas high SHAP values suggested an elevated probability of ICU mortality. We selected the median score (0.102) as the threshold for predicting low or high ICU mortality risk. For example, the first patient, who experienced ICU mortality, had a higher SHAP value and a prediction score of 0.543, indicating a higher risk of ICU mortality (**Figure 6D**). Conversely, the second patient, who did not experience ICU mortality, had a lower SHAP value and a prediction score of -0.332, indicating a lower risk of ICU mortality (**Figure 6E**). Since the higher the prediction score, the higher the probability of ICU mortality, we could use the model to distinguish between different survival probabilities and help clinical decision-making.

## Discussion

Because of the immunosuppression occurred in cancer patients, sepsis may begin and develop suddenly. The co-occurrence of lung cancer and sepsis presents significant challenges in diagnosis, treatment, and prognosis. Diagnostically, distinguishing between infection-induced systemic inflammatory response syndrome (SIRS) and tumor-related fever is complex, often leading to delays in appropriate therapy. Advanced imaging and biomarker analysis are essential but may be limited by the patient’s critical condition, calling a need for an outstanding biomarker to predict prognosis. In our study, we found that Oxford Acute Severity of Illness Score (OASIS) has the maximum predictability for ICU mortality in patients with sepsis and lung cancer. Several studies have compared OASIS with other severity scores such as Sequential Organ Failure Assessment (SOFA), Simplified Acute Physiology Score II (SAPS II), and Acute Physiology and Chronic Health Evaluation II (APACHE-II). A previous study demonstrated that OASIS, APACHE II, and SAPS II all presented good discrimination and calibration in predicting the 28-day mortality risk of acute kidney injury patients. OASIS, APACHE II, and SAPS II had better predictive accuracy than SOFA, but due to the complexity of APACHE II and SAPS II calculations, OASIS is a good substitute (26). Another study compared APACHE II, SOFA, SAPS II, and OASIS in predicting mortality in patients with sepsis or septic shock. The study found that all scoring systems were positively correlated with mortality, with SAPS II and OASIS showing higher correlations compared to others (27). These studies support our findings that OASIS is a robust predictor in the context of critical illness, which shed light on its predictive capabilities for septic patients with lung cancer.

Therapeutically, managing sepsis in lung cancer patients requires a delicate balance. Immunosuppressive effects of chemotherapy and the cancer itself increase susceptibility to infections, complicating sepsis management. Broad-spectrum antibiotics are standard; however, the potential for drug interactions and organ dysfunction necessitates careful selection and dosing. Recent studies have explored targeted therapies, such as aumolertinib, a third-generation EGFR-TKI, which has shown effectiveness in NSCLC cases with EGFR mutations (28). In the phase 3 AENEAS trial, aumolertinib significantly extended progression-free survival compared to gefitinib in patients diagnosed as advanced EGFR mutation-positive NSCLC (29). Prognostically, the combination of lung cancer and sepsis portends a poor outcome. Sepsis exacerbates the already compromised physiological state due to malignancy, leading to higher mortality rates. Early recognition and prompt, aggressive treatment of sepsis are crucial to improving survival (30).

Due to these challenges, accurate prediction of ICU mortality and identification of its risk factors are crucial for lung cancer patients with sepsis. The goal of our study is to establish a novel ML model for early ICU mortality prediction. By collecting essential clinic information and constructing ML models using a benchmark framework, we calculated risk scores for ICU mortality prediction, enabling precise prediction of ICU death probability. Once the risk tiers are established, the next step is to translating these into actionable clinical adjustments, which involves tailoring interventions based on the identified risk level. The clinical significance of this work is in enhancing patient management and therapy plan for patients with both lung cancer and sepsis, aiding clinicians in planning more informed, individual therapies. Moreover, the model’s predictions can assist in selecting adjuvant therapies, determining follow-up frequency, and deciding on additional lab tests. Incorporating this predictive model into clinic practices promotes data-driven decision-making, enhancing therapy outcomes and optimizing resource utilization. Ultimately, this integration helps standardize care across various healthcare providers and institutions, potentially decreasing diversity in treatment methods and therapy outcomes.

Besides, the key contribution of our study is the demonstration of how interpretable ML algorithms, particularly using SHAP values, can effectively identify critical factors influencing ICU mortality. The CatBoost algorithm, a gradient boosting framework based on symmetric decision trees (oblivious trees), excels in accuracy and efficiency, especially in handling categorical features, while requiring fewer parameters (31). Its performance often matches or surpasses that of other advanced ML algorithms, displaying outperforming discrimination, calibration, and clinical utility. However, due to its black-box nature, interpretation is essential for ML model. SHAP summary plots and force maps provide clinicians with clear, visual insights into the factors driving predictions, enhancing the model’s interpretability and highlighting key risk factors. Additionally, advanced ML techniques such as RFECV for feature selection, GridSearchCV for hyperparameter tuning, and SMOTE oversampling to address sample imbalance further improved the accuracy of ICU death prediction. This precise predictive model enables clinicians to develop personalized treatment strategies, ensuring timely interventions and improving the prognosis of lung cancer patients combined sepsis.

For critically sick patients, proactive and proactive treatment to address risk variables is essential (32). Nevertheless, some clinical variables are challenging to obtain in clinical practice, and many clinic variables show varying degrees of limitations in terms of accuracy, sensitivity, or specificity. Studies have indicated that SOFA scores often lack both sensitivity and specificity (33). Additionally, the clinical profiles and therapy outcomes of patients with both sepsis and lung carcinoma differ significantly from these patients with no cancer (34). Notably, the majority of critical illness scoring systems fail to consider cancer-specific factors (35). Specifically, we conducted univariate and multivariate logistic regression analyses to identify significant predictors of ICU mortality, including some cancer-related clinical factors. Leveraging these readily accessible clinic data, we successfully developed a robust CatBoost model for early ICU mortality prediction, thereby assisting clinicians in personalized therapy and decision-making. As observed in **Table 2**, there are some notable differences in the baseline profiles of patients among the training, internal testing and external testing databases, likely attributable to variations in hospital admissions. Despite these differences, the model demonstrated brilliant performances in both internal and external validation datasets, highlighting its robust applicability.

In our research, we observed that the presence of distant metastasis is linked to poor prognosis, likely due to the immunocompromised state of these patients (36). Immunosuppression has been shown to correlate with adverse outcomes in septic patients (37). Greater focus is needed on managing patients with distant metastasis to improve their outcomes. Older patients are at a higher risk of developing sepsis compared to younger individuals, and they often exhibit reduced resilience when managing the condition (38). Previous research has explored the association between the anion gap and prognosis across various diseases. As a well-established marker for evaluating acid–base balance (39), an abnormal anion gap is linked to acid–base disturbances, which are considered to significantly affect outcomes in critically sick patients (40). Similarly, our findings indicate that serum anion gap is a significantly risk variable for ICU mortality in patients with both sepsis and lung carcinoma. Several scoring systems for critical illness, including SAPS II, OASIS and SOFA scores, have been established to assess disease intensity and forecast short-term outcomes. The SAPS II is a scoring system developed to assess the intensity of illness in patients admitted to ICU (35). It evaluates 17 physiological variables, including vital signs and laboratory results, to generate a score that predicts the probability of hospital mortality, which was robust in our research for septic lung cancer patients’ mortality prediction. The OASIS is a prognostic tool to appraise the severity of intensity in critically sick patients. It incorporates variables such as age, heart rate, mean arterial pressure, temperature, respiratory rate, urine output, Glasgow Coma Scale, and specific laboratory values to generate a score that predicts in-hospital mortality (41), which was the most powerful indicator in our analysis for lung cancer patients’ mortality prediction, calling its application especially in lung cancer patients. The SOFA score is a clinical metric used to evaluate and quantify the degree of organ dysfunction across six physiological systems: respiratory, cardiovascular, hepatic, coagulation, renal, and neurological. It is particularly valuable in ICUs for monitoring disease progression, especially in sepsis cases (42), which was also validated in our analysis with septic lung cancer patients.

This study, while showcasing notable strengths, also has several limitations. First, we determined the required sample size for our external validation cohort. However, due to the limited availability of patients with complete follow-up data, we were unable to assemble a sufficiently large external validation set. While we acknowledge that larger sample sizes enhance the reliability of model evaluation, we have endeavored to utilize the maximum possible sample size given the current research constraints. To maximize the validation reliability despite the smaller external validation set, we employed a 10-fold cross-validation method to assess the model’s generalizability. Moving forward, we intend to expand the sample size of the external validation cohort in future research to further substantiate the model’s universality and reliability. Second, the study relies on retrospective information in the MIMIC IV database, which introduces the potential for selection bias. Variations in data collection across hospitals and the retrospective design also resulted in some missing clinical features. Additionally, the absence of key clinicopathological parameters, such as smoking, socioeconomic factors, and gene mutations, was a limitation, as the MIMIC IV database does not include imaging data. While we included a broad range of baseline and routine clinical features to improve predictive accuracy, this added complexity to the model’s practical use in clinical settings. Lastly, the model remains to be integrated into clinic practices, necessitating additional prospective, multicenter, and large-scale validation studies to confirm its applicability and practical utility in future settings.

## Conclusions

In our research, we successfully established a CatBoost-based prediction model using a ML benchmark framework to precisely forecast ICU mortality in lung carcinoma patients combined sepsis. We succeeded in identifying significantly predictive variables for ICU mortality in this patient population. This study establishes a groundwork for subsequent endeavors to refine ICU mortality predictions and prognostic forecasts, which may assist clinicians in making informed decisions and customizing therapeutic strategies.

## Data Availability

The raw data supporting the conclusions of this article will be made available by the authors, without undue reservation.

## Supplementary material

Supplementary Figure 1 Density plot of the variables containing missing values before and after imputation.

Supplementary Table 1 Comprehensive ranking of the clinic variables to predict ICU mortality in lung cancer patients combined sepsis base on RRA algorithm.
