# Supplementary material for: Personalized ICU mortality assessment by interpretable machine learning algorithms in patients with sepsis combined lung cancer: a population-based study and an external validation cohort
Source: Front Oncol. 2025 Oct 1;15:1661212. doi: 10.3389/fonc.2025.1661212 (PMC12522119; doi:10.3389/fonc.2025.1661212)

Supplementary Figure 1

Density plot of the variables containing missing values before and after imputation.
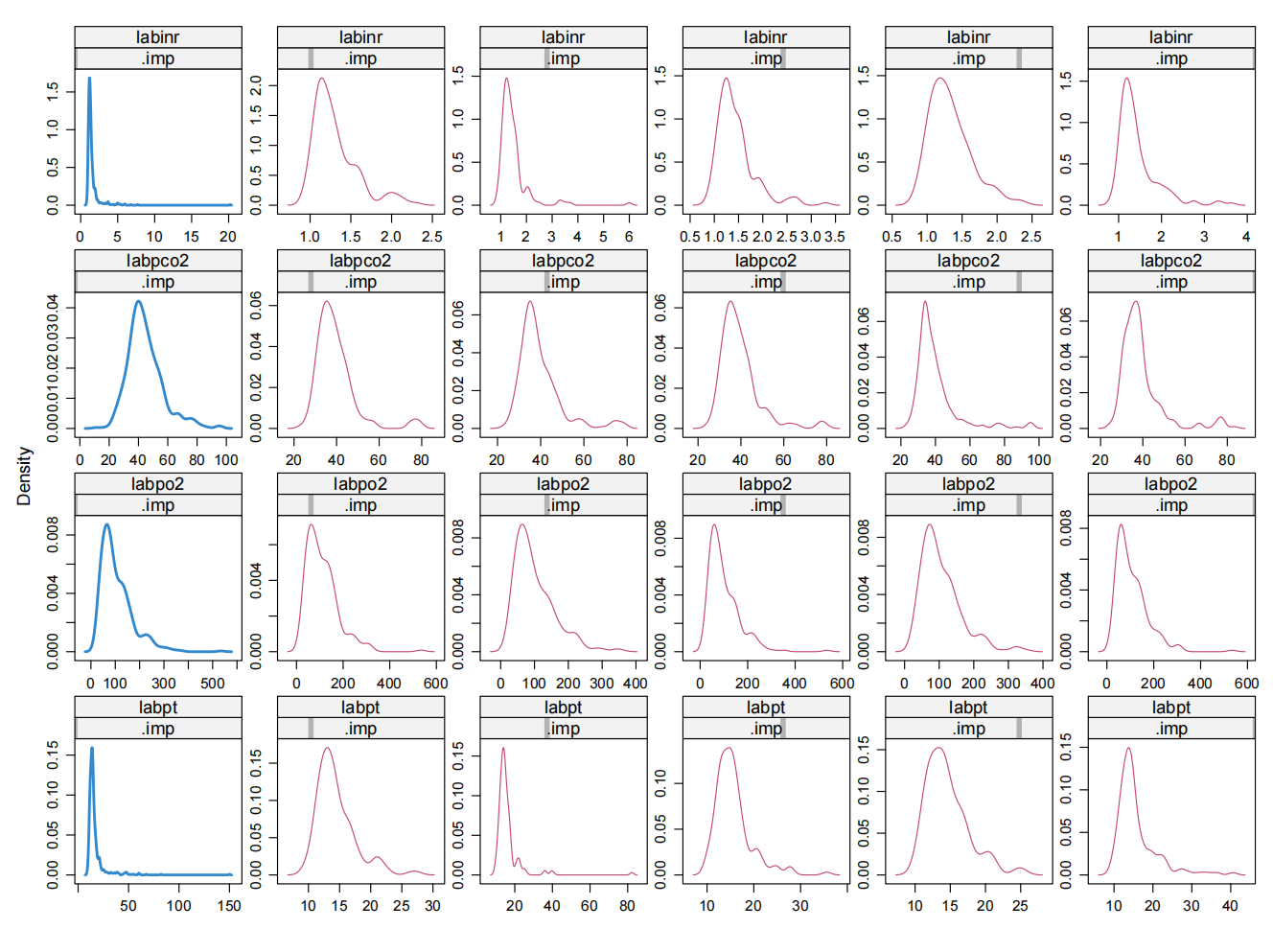

Supplement: Supplementary Figure 1 — Density plot of the variables containing missing values before and after imputation. [file DataSheet1.docx]
